# Factors influencing quality of life in low-income women with young children in Korea: a cross-sectional study

**DOI:** 10.4069/kjwhn.2022.03.03

**Published:** 2022-03-30

**Authors:** Yun Mi Kim, Ju-Hee Nho

**Affiliations:** 1 Department of Nursing, Hanil University and Presbyterian Theological Seminary, Wanju, Korea; 2 CCollege of Nursing, Research Institute of Nursing Science, Jeonbuk National University, Jeonju, Korea

**Keywords:** Child, Life style, Psychological distress, Quality of life, Women

## Abstract

**Purpose:**

This study aimed to investigate the effects of health-promoting behaviors (HPB), marital intimacy, and parenting stress on the quality of life (QoL) of low-income women with young children in Korea, an underserved group.

**Methods:**

This cross-sectional survey employed a descriptive correlational design. Using convenience sampling, 123 low-income women with children younger than 6 years were recruited from 14 health and community centers in Jeonju, Korea, from June 2020 to May 2021. Participants completed a questionnaire on QoL, HPB, marital intimacy, and parenting stress. Data were analyzed using descriptive statistics, independent t-test, analysis of variance, Pearson correlation, and hierarchical regression analysis.

**Results:**

Participants, who were on average 37.41±3.65 years old and had 1 to 2 children (n=98, 79.7%), reported a mid-level (3.14 out of 1–5) of QoL. Marital intimacy (β=.38, *p*<.001) was the most influential factor on the QoL of low-income women with young children. In descending order, HPB (β=.35, *p*<.001) and non-employment status (β=–.21, *p*=.003) had a significant influence on QoL (F=15.64, *p*<.001), and the overall explanatory power was 49.0%.

**Conclusion:**

Considering the mid-level QoL of low-income women with young children, programs aimed at improving the QoL of low-income women need to promote marital intimacy and maintain HPB, while considering their employment status. Strategies that include couple counseling, health care to encourage healthy lifestyles, and reemployment education are needed.

## Introduction

The incidence of poverty is an indicator of the state of communities and society, as well as critically affecting family and individual well-being. The Gini coefficient, representing income inequality, was 0.34 for Korea in 2020 ranking 11th out of 40 countries, and Korea’s employment rate was 67.2%, evaluated lower than Canada (74.8%) and the United States (70.5%) [1]. With the income gap widening as the number and rate of beneficiaries receiving basic livelihood security increases, continuous attention is required to prevent health inequality developing among those on low-income due to polarization.

Women play a key role in a family and their quality of life (QoL) will be more important when they are in a low-income situation. In a Hong Kong study comparing low-income households living in cities with their more affluent counterparts, the QoL of low-income households was poorer [2]. QoL of married working women differed in relation to income; the lower the income, the lower the QoL [3]. The QoL score of mothers caring for young children has also been reported as lower than that of women not caring for children [4,5]. In particular, low-income mothers with children have a lower QoL [4]; thus, strategies to identify and improve their QoL are needed. The relationship between the socioeconomic status of American mothers with child and the occurrence of cardiovascular disease and myocardial infarction shows that women’s economic status affected their health status [6]. In addition, in a study of 11,247 adults in Australia, lower education levels and lower income among women were related to higher levels of fasting insulin and triglycerides, and increased waist circumference, which contrasted with men [7]. Vulnerable women without insurance were also noted to have unhealthy lifestyle factors: 42% were smokers, 75% were overweight or obese, and approximately half had chronic disease risk factors [8]. These results suggest that low-income women struggle to maintain a healthy lifestyle in terms of their own physical health, and that their socially and economically vulnerable status often leads to greater exposure to lifestyle-related diseases. Factors such as alcohol consumption, smoking, and lack of exercise in women can significantly influence their QoL [9]. As such, for low-income women, health-promoting behaviors (HPB) need to be considered as a major variable affecting QoL in relation to health maintenance.

For women in marital relationships, marital intimacy has been shown to be positively correlated with QoL [10], in areas such as communication, mutual respect, sexual life, leisure activities, marital satisfaction, and emotional expression. However, for low-income couples, trying to deal with economic pressure can hinder coping with conflict and expressing intimacy in marital relationships [11]. Therefore, marital intimacy can be considered as a variable that has a major influence on women’s QoL in terms of relational aspects.

Stress is also a major influencing factor on QoL of adult women [12]. Specifically, parenting stress is an important factor in mothers’ health management during the period of marriage, pregnancy, and childbirth [13-15]. Through marriage, marital intimacy, parenting, and personal aspects of life can exert influence, and parenting stress due to maternal role demands is reported as one of the risk factors in the family environment among low-income mothers [16].

Despite the high importance of HPB, marital intimacy, and parenting stress among low-income women with children, studies on low-income women caring for young children in Korea are lacking. The purpose of this study was to identify the HPB, marital intimacy, parenting stress, and QoL of low-income women with young children, and to examine the influencing factors on QoL. The specific research objectives were as follows: (1) determine the levels of and relationships among HPB, marital intimacy, parenting stress, and QoL, (2) identify QoL according to participant characteristics, and (3) examine the influencing factors on QoL.

## Methods

Ethics statement: This study was approved by the Institutional Review Board of Jeonbuk National University (No. 2020-05-006-004). Informed consent was obtained from the participants.

### Study design

This study used a descriptive correlational research design aimed to identify factors affecting QoL in low-income women with young children through a cross-sectional survey. This study was described in accordance with the STROBE (Strengthening the Reporting of Observational Studies in Epidemiology) guidelines (https://www.strobe-statement.org).

### Participants

The participants of this study were low-income women with young children. The inclusion criteria were as follows: (1) medical benefit recipients or basic livelihood recipients with a monthly household income below 50% of the South Korean national median income, i.e., about 2.4 million Korean won (four persons) for 2020 (approximately 1,900 US dollars) [17]; (2) women with children aged less than 6 years; and (3) those understanding the purpose of the study and agreeing to participate. The exclusion criteria were as follows: (1) women currently pregnant, (2) having a psychiatric condition or on medication, (3) being in a single-parent family or a multicultural family, (4) women without spouses, and (5) having a child requiring long-term care, such as developmental diseases.

### Study size

The G*Power 3.1.9 program [18] was used to calculate the appropriate number of participants required, based on power (1-β) .80 and a median effect size of .15 based on previous studies [19,20], and significance level (α) of .05. When 11 predictors (age, education level, employment status, monthly income, religion, type of family, number of children, husband’s parental attitude, HPB, marital intimacy, and parenting stress) were input, a minimum of 123 participants were required. Questionnaires were distributed to 150 women considering a dropout rate of 20%, and data from 123 participants (82.0%) were analyzed after excluding refusals (n=17), incomplete data (50% or more blank, n=3), and ineligible cases (no children, n=7).

### Setting and data collection

Participant recruitment and data collection were conducted from June 6, 2020 to May 6, 2021. The researchers advertised study recruitment at two health centers and 12 community centers in Jeonju, Korea. Considering the vulnerable status of participants it was explained that participants could withdraw from the study at any time, the collected data would be used only for research, and that confidentiality of personal information would be guaranteed. Participants filled out the self-report questionnaire in an office at the center or cafe where privacy was maintained. The questionnaire took approximately 20 to 30 minutes and a gift certificate (worth 4 US dollars) was given as a token of appreciation.

### Measurements

#### Quality of life

QoL was measured using the Korean version of the World Health Organization Quality of Life, brief version (WHOQOL-BREF) [21]. This instrument has 26 questions consisting of four domains: physical health (seven items), psychological health (six items), social relationships (three items), and environment (eight items); and includes one item each for overall QoL and general health. Following the Korean version manual [21], each item was rated on a 5-point Likert scale (‘not at all,’ 1 to ‘all the time,’ 5), with higher mean scores (possible range, 1–5) indicating higher QoL. The subscale scores are calculated by multiplying the mean score of each subscale by 4 and the possible score range is 4 to 20 points. The Cronbach’s α of the Korean version was .90 [21], and .90 in our study. We obtained permission to use the Korean version of WHOQOL-BREF prior to its use.

#### Health-promoting behaviors

HPB was measured using the Korean version [22] of the Health-Promoting Lifestyle Profile-II (HPLP-II) developed by Walker et al. [23] after obtaining permission. This measurement consists of a total of six subdomains and 52 items, including for health responsibility (nine items), physical activity (eight items), nutrition (nine items), spiritual growth (nine items), interpersonal relationships (nine items), and stress management (eight items). Each item is scored on a 4-point Likert scale (‘not at all,’ 1 to ‘all the time,’ 4); and the higher the summed score (possible range, 52–208), the more positive the response to the HPB. The Korean version of the HPLP-II has well-established validity and reliability [22]. Cronbach’s α coefficients for the total score were .93 in the Korean version [22], and .94 in the current study.

#### Marital intimacy

Marital intimacy was measured using the 15-item Marital Intimacy tool [24], which was developed in Korean. The subdomains (five items each) consist of cognitive, emotional, and sexual intimacy and items are assessed using a 5-point Likert scale (‘not at all,’ 1 to ‘strongly agree,’ 5). Higher summed scores (possible range, 15–75) indicate greater marital intimacy. At the time of development, Cronbach’s α was .90; and in this study, it was .86. Permission was obtained prior to use.

#### Parenting stress

The Korean version of the Parenting Stress Index-Short Form-4th edition (PSI-SF-4) [25] was purchased (https://inpsyt.co.kr/psy/item/view/KPSI4_CO_PG) and used. The PSI-SF-4 consists of 36 items in three subdomains: parental distress, parent-child dysfunctional interaction, and difficult child. Each item is rated on a 5-point Likert scale (‘strongly disagree,’ 1 to ‘strongly agree,’ 5), and higher summed scores (possible range, 36–180) indicate higher parenting stress. The Korean version of the PSI-SF-4 had Cronbach’s α of .96 [26]; and in the current study, it was .92.

#### General characteristics

General characteristics included age, education level, employment status, monthly income, religion, type of family, number of children, and husband’s parental attitude (passive, moderate, or active).

### Data analysis

The collected data were statistically processed using IBM SPSS ver. 25.0 (IBM Corp., Armonk, NY, USA). Descriptive statistics were done and variations in QoL according to general characteristics were analyzed using independent t-tests and one-way analyses of variance followed by the Scheffé test. Pearson correlation analysis was done to identify the relationships between QoL and other variables and hierarchical regression analysis was used to investigate influencing factors on QoL.

## Results

### Participants’ general characteristics and quality of life according to general characteristics

Participants’ mean age was 37.41 years, 74 (60.2%) had a bachelor degree or more and 65 (52.8%) were currently unemployed. Most participants (n=107, 87.0%) had a mean monthly household income between 2 million and 3 million Korean won (approximately 1,800-2,700 US dollars). Having two children was most common (n=67, 54.5%) and five participants (4.1%) had four or more. As for the husband’s parental attitude, 87.8% showed an attitude ‘moderate or active.’

Participants with a university degree or higher had significantly higher QoL than those who had a high school education or less (F=4.51, *p*=.013), and employed women had significantly higher QoL than unemployed women (t=3.37, *p*<.001). In addition, the levels of QoL were statistically significantly different according to husband’s parental attitude (F=5.50, *p*=.005) (Table 1).

#### Levels of quality of life, health-promoting behaviors, marital intimacy, and parenting stress

The mean score of QoL was midpoint at 3.14, with the highest sub-score noted in physical health QoL (13.01±2.41). HPB was close to midpoint at 120.56 and marital intimacy was greater than midpoint at 50.38. Parenting stress was relatively low at 77.17 (Table 2).

#### The relationships among quality of life, health-promoting behaviors, marital intimacy, and parenting stress

QoL showed a positive moderate correlation with HPB (r=.57, *p*<.001) and marital intimacy (r=.56, *p*<.001), and a negative weak correlation with parenting stress (r=–.23, *p*=.010). For HPB, a positive moderate correlation with marital intimacy (r=.40, *p*<.001) and a negative weak correlation with parenting stress (r=–.25, *p*=.005) was also noted (Table 3).

#### Factors influencing quality of life

For hierarchical regression analysis, based on previous studies [9,10,12,16,27,28], the main variables were entered as the first model for general characteristics that showed differences in QoL, the second model for HPB as an individual factor, the third model for marital intimacy as relationship with spouse, and the fourth model for parenting stress as family relationship. To confirm the residual normality, the residual histogram, residual normal probability graph, and residual homoscedasticity graph were checked, and the results were found to be satisfactory. Cook’s distance was less than 1.0, at 0.00 to 0.17; thus, no cases needed to be deleted. The Durbin-Watson value was close to 2, at 2.12, indicating independence between individuals. The variance inflation factor values were less than 10, at 1.09 to 3.36, and the tolerance ranged from 0.29 to 0.92, which was more than 0.1, confirming no multicollinearity between the variables, and that the conditions for regression analysis were satisfied.

The dummy variables were educational level (≤high school), occupation (employed), and husband’s parental attitude (passive), based on the findings in Table 1. Model 1 showed that general factors explained 17.0% of the variance in QoL in low-income women with a young child. In model 2, HPB (β=.49, t=6.56, *p*<.001) significantly influenced QoL and the explanatory power increased to 39.0%. In model 3, the explanatory power increased to 49.0% and marital intimacy (β=0.38, t=5.08, *p*<.001) exerted the greatest influence on QoL, followed by HPB (β=.35, t=4.72, *p*<.001) and employment (β=–0.21, t=–3.08, *p*=.003). In model 4, although parenting stress (β=–.01, t=–.20, *p*=.844) did not significantly influence QoL, marital intimacy (β=.38, t=4.88, *p*<.001), HPB (β=.35, t=4.62, *p*<.001) and employment (β=–.21, t=–3.07, *p*=.003) were significant influencing factors.

Thus, the total explanatory power of QoL in low-income women with young children was thus 49.0% in the hierarchical regression model (F=15.64, *p*<.001), with three explanatory variables: occupation (unemployed), HPB, and marital intimacy (Table 4).

## Discussion

The level of QoL in this study was difficult to directly compare to prior studies, as few used the same instrument in vulnerable participants with similar demographic characteristics. In this study, the QoL subdomain scores of low-income women with preschool children were 12 to 13 points out of a possible range of 4 to 20, with the lowest scores for environmental, social relationships, and psychological health domain. It is lower than the QoL reported for low-income women in China using the 36-item Short Form survey, which identified 60 to 70 points out of 100 for QoL subdomains [19]. As low-income participants may be vulnerable in terms of psychological and social relationships, with multiple issues consequently arising, this underscores the need for closer attention to and provision of support concerning the relationship and psychological needs of low-income women, especially when rearing young children.

The greatest influencing factor on QoL was marital intimacy in this study. This is consistent with a previous study that marital intimacy was an important influencing factor on the QoL of Korean women [27]. In addition, poor marital of low-income Chinese mothers caring for young children has been reported as a negative effect on mothers’ QoL, which had a negative effect on their children’s QoL and behavior [29]. Another study in Korea [30] reported that low-income couples with high economic pressure experience greater marital conflict than couples with general income levels. Therefore, to improve the QoL of low-income women with young children, effective marital intimacy promotion programs, such as relationship improvement education and counseling programs, should not be overlooked for family health.

In this study, HPB was also identified as an important factor influencing QoL. This finding supports a previous study’s report that women with better HPB demonstrated better QoL [12]. Low-income women have less access to fresh and healthy foods and they consume a lot of soft drinks (soda/cola), fructose-containing drinks, and fast food [31]. In addition, access to exercise facilities is poor, and there are few opportunities to use exercise equipment or gymnasiums, leading to low physical activity and overall low level of HPB [32]. These unhealthy HPB affect not only low-income women but also the health of their children and families [16]. Thus, nurses can offer educational support for low-income women to adopt healthy behaviors in terms of food intake and physical activity. HPB interventions such as lifestyle modification program should be planned and delivered to improve the health of low-income women with young children.

In this study, parenting stress had a weak negative correlation with QoL but was not a significant factor affecting QoL. Most participants in this study were younger than 40 years of age, 52.8% were unemployed, and 87.8% of their spouses favorably supported participation in parenting, which may explain the relatively low score level. This is similar to a previous study of low-income women in the US that showed young age, public support, and spousal support having a positive relationship with QoL [16]. More studies are needed to further determine the seemingly negative relationship with economic activity and women’s QoL Given that data collection occurred during the COVID-19 pandemic when caring for young children at home may have affected perceptions of parenting stress compounded by general concerns, large-scale research that considers various stages of child growth and development and types of economic stress is required.

The QoL of the women in this study showed significant differences according to their education level and their spouses’ parenting participation. In particular, findings were consistent with prior studies that showed that women with more formal education reported higher QoL and that the QoL of working women was higher [6,7]. A higher QoL may be linked to education level, women’s social activities in terms of their occupation. Among married Korean women, higher QoL has been found when easy marital communication occurs and when women are highly educated and have greater socioeconomic status [33]. For employed women raising young children, the lower the child support cost, the lower levels of QoL were found [34]. These results indicate that women’s economic activities and levels of support affect their QoL. As found in this study, the spouse’s active attitude to participate in parenting can help women try to balance the economic status related to parenting and work and family. Also, efforts such as education and reemployment training, public health services, and couple counseling activities should be made to improve the QoL of low-income women with young children.

This study has a limitation that only parenting stress was examined as a specific psychological factor and other factors such as individual depression and anxiety were not measured. Convenience sampling from one region in Korea may also limit its generalizability. However, this study is the first to our knowledge, to assess physical, psychological, and relational factors that influence QoL in low-income Korean women rearing preschool-aged children. As such, it provides empirical data on and can inspire interest in the lives of low-income women. By identifying the effects of general characteristics, HPB, marital intimacy, and parenting stress on the QoL of low-income women, better targeted practical intervention strategies can be developed in the nursing field and in government policies to foster healthier lifestyles among low-income women. Understanding issues affecting the QoL of life of low-income women with young children and implementing appropriate intervention strategies may have a positive effect on family planning related to childbirth in an era of low fertility. Encouraging healthier lifestyles among low-income women raising children can be expected to have a positive effect on all family members. In this regard, further studies that focus on program development to improve the QoL of low-income women and verify its effectiveness are recommended.

In conclusion, the QoL of low-income Korean women with young children was found to be mid-level, and marital intimacy, HPB, and employment status explained 49% of the variance. Based on the results of this study, it is recommended that efforts be made to enhance marital intimacy and effective HPB while considering their employment status. In addition, strategies through community self-reliance centers, reemployment education, and reinforcing basic health care are necessary. Nurses can participate in promoting healthy lifestyles, marital intimacy, and expanding career opportunities through a multidisciplinary approach to support women from low-income families caring for young children.

## Figures and Tables

**Table 1.** Quality of life by participants’ characteristics (N=123)

Variable	Categories	Mean±SD or n (%)	Quality of life Mean±SD	t/F (*p*)
Age (year)		37.41±3.65		
	<30	2 (1.6)	3.60±0.52	1.40 (.266)
	30-39	83 (67.5)	3.10±0.52	
	≥40	38 (30.9)	3.21±0.57	
Education	≤High school^a^	11 (8.9)	2.84±0.54	4.51 (.013)
	College^b^	38 (30.9)	3.01±0.56	a<c^†^
	≥University^c^	74 (60.2)	3.25±0.50	
Employment status	Employed	58 (47.2)	3.31±0.49	3.37 (.001)
	Unemployed	65 (52.8)	3.00±0.54	
Monthly income (KRW)^‡^	<1 million	5 (4.1)	2.76±0.47	2.00 (.139)
	1–2 million	11 (8.9)	2.98±0.46	
	2–3 million	107 (87.0)	3.18±0.54	
Religion	Yes	74 (60.2)	3.18±0.55	0.91 (.364)
	No	49 (39.8)	3.09±0.53	
Type of family	Nuclear family	113 (91.9)	3.14±0.55	0.06 (.957)
	Large family	10 (8.1)	3.13±0.50	
Number of children		1.99±0.73		
	1	31 (25.2)	3.11±0.45	0.12 (.949)
	2	67 (54.5)	3.13±0.55	
	3	20 (16.3)	3.20±0.69	
	≥4	5 (4.1)	3.18±0.40	
Husband’s parental attitude	Passive	15 (12.2)	2.75±0.55	5.50 (.005)
	Moderate	59 (48.0)	3.15±0.47	
	Active	49 (39.8)	3.26±0.57	

KRW: Korean won (1 million KRW is approximately 900 US dollars).

† Scheffé test.

**Table 2.** Levels of quality of life, health-promoting behaviors, marital intimacy, and parenting stress (N=123)

Variable	Categories	Mean±SD	Reported range	Possible score range
Quality of life		3.14±0.54	1.58–4.42	1–5
	Physical health	13.01± 2.41	6–18	4–20
	Psychosocial health	12.44± 2.63	4–18	4–20
	Social relationships	12.30± 2.95	6–18	4–20
	Environmental	12.24± 2.58	6–19	4–20
Health-promoting behaviors		120.56±19.69	74–175	52–208
	Health responsibility	19.23±4.13	10–31	9–36
	Physical activity	14.13±4.79	8–30	8–32
	Nutrition	22.32±4.29	12–36	9–36
	Spiritual growth	22.48±4.91	11–33	9–36
	Interpersonal relations	24.54±3.99	16–36	9–36
	Stress management	17.87±4.10	9–32	8–32
Marital intimacy		50.38±9.26	23–71	15–75
	Cognitive	17.27±3.56	5–25	5–25
	Emotional	16.59±3.07	8–23	5–25
	Sexual	16.52±4.15	5–25	5–25
Parenting stress		77.17±16.65	45–150	36–180
	Parental distress	32.85±7.79	13–57	12–60
	Parent-child dysfunctional interaction	18.68±6.40	12–51	12–60
	Difficult child	25.63±6.26	14–47	12–60

**Table 3.** Relationships among quality of life, HPB, marital intimacy, and parenting stress (N=123)

Variable	r (*p*)		
	Quality of life	HPB	Marital intimacy
Quality of life	1		
HPB	.57 (<.001)	1	
Marital intimacy	.56 (<.001)	.40 (<.001)	1
Parenting stress	–.23 (.010)	–.25 (.005)	–.33 (<.001)

HPB: Health-promoting behaviors.

**Table 4.** Factors influencing quality of life (N=123)

Factor	Model 1		Model 2		Model 3		Model 4	
	β	t (*p*)	β	t (*p*)	β	t (*p*)	β	t (*p*)
Education†								
College or less	.21	1.40 (.164)	.09	.70 (.484)	.13	1.15 (.254)	.14	1.15 (.251)
≥University	.37	2.52 (.013)	.16	1.24 (.219)	.22	1.82 (.071)	.22	1.82 (.071)
Occupation†	–.24	–2.77 (.007)	–.20	–2.75 (.007)	–.21	–3.08 (.003)	–.21	–3.07 (.003)
Husband’s parental attitude†								
Moderate	.37	2.73 (.007)	.27	2.26 (.026)	.17	1.58 (.118)	.17	1.58 (.117)
Active	.38	2.80 (.006)	.28	2.37 (.019)	.10	.89 (.375)	.10	.89 (.378)
Health-promoting behaviors			.49	6.56 (<.001)	.35	4.72 (<.001)	.35	4.62 (<.001)
Marital intimacy					.38	5.08 (<.001)	.38	4.88 (<.001)
Parenting stress							–.01	–.20 (.844)
R^2^	.20		.42		.52		.52	
Adjusted R^2^	.17		.39		.49		.49	
F (*p*)	5.84 (<.001)		13.77 (<.001)		18.02 (<.001)		15.64 (<.001)	
ΔAdjusted R^2^			.22		.10		0	

† The reference variables were education (≤high school), occupation (employed), and husband’s parental attitude (passive).
